# Automatic segmentation of male pelvic anatomy on computed tomography images: a comparison with multiple observers in the context of a multicentre clinical trial

**DOI:** 10.1186/1748-717X-8-106

**Published:** 2013-04-30

**Authors:** John P Geraghty, Garry Grogan, Martin A Ebert

**Affiliations:** 1 Department of Radiation Oncology, Sir Charles Gairdner Hospital, Perth, Western Australia, Australia; 2 School of Physics, University of Western Australia, Perth, Western Australia, Australia

**Keywords:** Computed tomography, Segmentation, Volume, Clinical trials, Automation, Quality assurance

## Abstract

**Background:**

This study investigates the variation in segmentation of several pelvic anatomical structures on computed tomography (CT) between multiple observers and a commercial automatic segmentation method, in the context of quality assurance and evaluation during a multicentre clinical trial.

**Methods:**

CT scans of two prostate cancer patients (‘benchmarking cases’), one high risk (HR) and one intermediate risk (IR), were sent to multiple radiotherapy centres for segmentation of prostate, rectum and bladder structures according to the TROG 03.04 “RADAR” trial protocol definitions. The same structures were automatically segmented using iPlan software for the same two patients, allowing structures defined by automatic segmentation to be quantitatively compared with those defined by multiple observers. A sample of twenty trial patient datasets were also used to automatically generate anatomical structures for quantitative comparison with structures defined by individual observers for the same datasets.

**Results:**

There was considerable agreement amongst all observers and automatic segmentation of the benchmarking cases for bladder (mean spatial variations < 0.4 cm across the majority of image slices). Although there was some variation in interpretation of the superior-inferior (cranio-caudal) extent of rectum, human-observer contours were typically within a mean 0.6 cm of automatically-defined contours. Prostate structures were more consistent for the HR case than the IR case with all human observers segmenting a prostate with considerably more volume (mean +113.3%) than that automatically segmented. Similar results were seen across the twenty sample datasets, with disagreement between iPlan and observers dominant at the prostatic apex and superior part of the rectum, which is consistent with observations made during quality assurance reviews during the trial.

**Conclusions:**

This study has demonstrated quantitative analysis for comparison of multi-observer segmentation studies. For automatic segmentation algorithms based on image-registration as in iPlan, it is apparent that agreement between observer and automatic segmentation will be a function of patient-specific image characteristics, particularly for anatomy with poor contrast definition. For this reason, it is suggested that automatic registration based on transformation of a single reference dataset adds a significant systematic bias to the resulting volumes and their use in the context of a multicentre trial should be carefully considered.

## Background

Inter-observer variation in anatomical segmentation ^a^ remains as one of the primary limitations to high geometric accuracy in radiotherapy [1,2]. Simultaneously, extensive and accurate anatomical segmentation is becoming central to the radiotherapy treatment planning environment. The computationally-intensive methods utilised by inverse treatment planning are driven by defined anatomical volumes, and four-dimensional, multi-modal and sequential imaging approaches (for adaptive radiotherapy) require multiple segmentations on multiple image data sets for a single patient.

Multicentre clinical trials involve a collation of the multiple variables contributing to inter-observer segmentation variation. These variables include the observers themselves, their experience, the influence of local practice, and the platforms on which they undertake such segmentation. In the context of a multicentre trial, participating centres are contributing data that incorporates these variables, together with the influence of interpretation of anatomical volumes as defined by a trial protocol. Frequently, this data will be incorporated into trial outcomes analyses, and subsequently translated to guiding future patient treatments. One way of assessing inter-observer variations in segmentation is via a ‘dummy-run’ or ‘benchmarking study’ undertaken at the commencement of the trial, whereby sample patient radiographic information is distributed to participating centres, anatomy segmented as per protocol definition, and the resulting contours sent back to the trial coordinator for assessment (see for example, Matzinger *et al.*[3]).

One strategy to homogenise anatomical segmentation is to employ computer-based algorithms for automatic segmentation of relevant patient anatomy, based either on image-feature detection methods, or established anatomical atlases (‘automatic segmentation’) [4-6]. Although offering significant potential for consistency in outlining approach within and between patients, the structures such algorithms generate will be dictated by the data used in algorithm development and could be inconsistent with anatomical structures defined by human observers.

With the potential for high geometric accuracy through image-guidance, possibly using daily imaging, with well-defined adjacent normal tissues, combined with poor image specificity on computed tomography (CT) images, the prostate makes a suitable candidate for automatic segmentation techniques. Issues associated with prostate definition on CT have been well documented, especially at prostatic base and apex [7-10]. The question is posed therefore if automatic segmentation techniques could be utilised to homogenise definition of the prostate and associated anatomy across the population of participants contributing to a multicentre clinical trial, with the potential to enhance the translatability of outcomes analyses [11].

We had available multi-observer outlines for prostate and normal tissues for sample prostate radiotherapy patients, collected via a benchmarking study undertaken in support of a multicentre clinical trial of prostate radiotherapy across Australia and New Zealand. A comparison was undertaken between these outlines and those generated by the pelvic-anatomy automatic segmentation algorithm of a commercial treatment planning system (TPS). The aim was to identify systematic variations between this algorithm and the observers.

## Methods

### Benchmarking data

As part of quality assurance (QA) methods for the TROG 03.04 RADAR (Randomized Androgen Deprivation and Radiotherapy, NIH trial identifier NCT00193856) [12,13], CT images (‘reference’ images) for two patients were electronically distributed to participating centres where treatment plans were to be generated based on the trial protocol. The segmentation requirements from the RADAR protocol are summarised in Table 1. One set of images was for an ‘intermediate risk’ (IR) patient (defined as TNM classification < 3b; Gleason score < 8; prostate-specific antigen (PSA) score < 20 ng/ml), and the other for a ‘high risk’ (HR) patient (defined as TNM classification ≥ 3b; Gleason score ≥ 8; PSA ≥ 20 ng/ml). Images were axial scans, 3 mm slice thickness at 512 × 512 resolution. Once generated, treatment plans were to be digitally exported in either RTOG [14] or DICOM-RT [15] format. From this exercise, 11 HR patient plans and 7 IR patient plans were available for this particular study, together with the original patients’ DICOM images, retrieved from 13 participating centres across Australia and New Zealand. Segmentation on the plans was performed manually by the local participating clinician in each case using locally-available software tools, with no guidance provided on image window/level settings or segmentation technique.

**Table 1:** Requirements for segmentation of target volumes, rectum and bladder for the TROG 03.04 RADAR trial

**Structure**	**IR Definition**	**HR Definition**
Gross target volume (GTV)	Prostate + extra capsular extension	Prostate + seminal vesicles + extra capsular extension
Clinical target volume (CTV)	CTV = GTV	CTV = GTV
Rectum	The outer wall of the rectum from the cranial border (where the rectum turns horizontally into the sigmoid colon), to the caudal border (defined as 15 mm caudal to the apex of the prostate)	
Bladder^a^	Visible bladder	

^a^Note that in the RADAR trial, segmentation of bladder was only a requirement for a sub-set of patients.

The reference images were also imported into the iPlan TPS (Brainlab AG, Feldkirchen, Germany), version 1.5.0 (build 118064) and GTV/CTV, rectum and bladder segmented using the iPlan RT Automatic Atlas Segmentation algorithm. This algorithm creates a deformation field to map an established atlas to the patient’s images. This is achieved by determining the transformation vector at each point in the reference image set (used to define the segmentation atlas) required to map that point to the equivalent one in the patient image set, in order to maximise similarity measures. The deformation map is applied to anatomical volumes defined in the atlas by mapping points defining those volumes according to the established transformation vectors. The subsequently deformed volumes can then be added to the patient images [16,17]. Following application of this algorithm on the sample treatment plans, the images and mapped structure definitions were exported from iPlan in DICOM-RT format.

The resulting 18 observer-segmented datasets and 2 iPlan-segmented datasets were imported into the SWAN system [18] and data describing the relevant 3D structures exported as comma-separated values (CSV) files. These were able to be read by the VAST software tool [19]. The process of import of reference images into the local TPS at each centre and then re-export frequently introduces a coordinate system translation and/or inversion. With the assistance of external radio-opaque fiducials on the reference image set it was possible to quantify these changes and correct for them in VAST to re-align the coordinates defining all structures. Structures could then be saved in an extensible markup language (XML) format with parameters included to describe the coordinate transformations. For structures from iPlan for the HR case, a single CTV was formed by union (in SWAN) of the independent prostate and seminal vesicle structures.

Structure volume (for observer-defined structures, V_Obs_, and iPlan-defined structures, V_iPlan_), calculated in SWAN was used as a single quantitative measure for comparison of the structures. Due to the convoluted and often convex nature of the structures, centre-of-mass was not considered a valid quantity for the comparison. The percentage difference of the observer-defined relative to the iPlan-defined volume, $\Delta V_{\mathrm{iPlan}}^{\mathrm{Obs}}$, was calculated for each structure according to:

(1) $$\Delta V_{\mathrm{iPlan}}^{\mathrm{Obs}}=100\times \frac{\left(V_{\mathrm{Obs}}-V_{\mathrm{iPlan}}\right)}{V_{\mathrm{iPlan}}}.$$

A structure representing the intersection of each observer-defined and iPlan-defined anatomical structure was obtained in SWAN. This structure has volume *V*_*Obs*∩*iPlan*_ and was used to calculate the DICE similarity coefficient (DSC) [20] according to:

(2) $$\mathrm{DSC}=2\times \frac{V_{\mathrm{Obs}\cap \mathrm{iPlan}}}{V_{\mathrm{Obs}}+V_{\mathrm{iPlan}}},$$

as well as the volume of intersection relative to the observer-defined volume, *I*_*Obs*_^*Obs*∩*iPlan*^:

(3) $$I_{\mathrm{Obs}}^{\mathrm{Obs}\cap \mathrm{iPlan}}=\frac{V_{\mathrm{Obs}\cap \mathrm{iPlan}}}{V_{\mathrm{Obs}}}.$$

The spatial variation in agreement between the multiple observers and iPlan-defined structures was quantified by determining the mean distance between each point comprising the iPlan-defined structure on each image slice, and the multiple observer defined structures on that slice and each adjacent slice. This allowed generation of a surface map of iPlan and observer agreement, projected onto the iPlan-defined structure. For details of the calculation method, implemented in the VAST software tool, see Ebert *et al.*[19].

### Sample trial data

20 trial participant datasets were randomly selected from the full set of 754 archived participant plans. This sample included plans for 11 IR and 9 HR patients, though subsequent analysis does not discriminate these two groups. Images for each plan included axial slices ranging in thickness from 2.5 to 5.0 mm. Plans were selected without knowledge of their origin and so the relationship of the observers to those who contributed the 18 benchmarking plans is not known. Each of the 20 sample trial plans was imported into iPlan and bladder, rectum and CTV segmented using the automatic segmentation algorithm. The resulting structures were exported in DICOM format. Each original plan was uploaded in SWAN together with the iPlan-defined structures for comparison with the equivalent observer-defined structures, assessed via the parameters defined above - V_Obs_, V_iPlan_, $\Delta V_{\mathrm{iPlan}}^{\mathrm{Obs}}$, *V*_*Obs*∩*iPlan*_, DSC, and *I*_*Obs*_^*Obs*∩*iPlan*^. Additionally, the extent along the superior-inferior (Z) axis ^b^ of each observer-defined structure was compared to the equivalent iPlan-defined structure. This was achieved by calculating the distance between the most superior slices where each structure is defined, *z*_*Sup*,*Obs*_ − *z*_*Sup*,*iPlan*_, and the distance between the most inferior slices where each structure is defined, *z*_*Inf*,*Obs*_ − *z*_*Inf*,*iPlan*_. Given the coordinate system on the associated images, a positive value indicates an observer’s structure being on a more superior slice. Results of this analysis are presented as the average across the 20 sample datasets. Note that, as segmentation of bladder was not compulsory under the RADAR protocol, only 14 of the 20 sample plans had a bladder outlined.

## Results

### Benchmarking data

Table 2 and Table 3 summarise the quantitative parameters for each of the structures for the HR and IR reference patients respectively, as segmented by all observers and as automatically segmented in iPlan, together with the comparative measures based on structure intersection. Note some missing and incomplete data for the bladder as bladder segmentation was not a requirement of the RADAR trial.

**Table 2:** Summary of quantitative measures for structures for the HR case benchmarking study patient

**CTV**					
**Observer**	V_iPlan_ (cm^3^)	$\Delta V_{\mathrm{iPlan}}^{\mathrm{Obs}}$ (%)	*V*_*Obs*∩*iPlan*_ (cm^3^)	*DSC*_*Obs*_^*iPlan*^	*I*_*Obs*_^*Obs*∩*iPlan*^
***iPlan***	*54.0*	*-*	*-*	*-*	*-*
**H**	50.2	−6.9	40.3	0.77	0.80
**A**	75.2	39.4	51.0	0.79	0.68
**I**	60.0	11.2	48.0	0.84	0.80
**B**	84.3	56.3	50.8	0.74	0.60
**C**	41.2	−23.6	35.8	0.75	0.87
**D**	87.0	61.2	52.1	0.74	0.60
**E**	78.6	45.6	46.2	0.70	0.59
**F**	70.8	31.2	47.6	0.76	0.67
**J**	158.8	194.3	54.0	0.51	0.34
**K**	82.5	53.0	48.8	0.72	0.59
**L**	62.8	16.4	47.1	0.81	0.75
***Mean A-F, H-L (SD)***	*77.4 (30.7)*	*43.5 (56.8)*	*47.4 (5.3)*	*0.74 (0.09)*	*0.66 (0.15)*
**BLADDER**					
**Observer**	V_iPlan_ (cm^3^)	$\Delta V_{\mathrm{Obs}}^{\mathrm{iPan}}$ (%)	*V*_*Obs*∩*iPlan*_ (cm^3^)	*DSC*_*Obs*_^*iPlan*^	*I*_*iPlan*_^*Obs*^
***iPlan***	*160.3*	*-*	*-*	*-*	*-*
**H**	-^a^	-^a^	-^a^	-^a^	-^a^
**A**	172.9	7.9	153.4	0.92	0.89
**I**	152.6	−4.8	147.6	0.94	0.97
**B**	165.7	3.4	151.5	0.93	0.91
**C**	157.8	−1.6	146.9	0.92	0.93
**D**	158.2	−1.3	145.8	0.92	0.92
**E**	164.4	2.6	152.3	0.94	0.93
**F**	149.5	−6.7	140.0	0.90	0.94
**J**	184.9	15.3	153.1	0.89	0.83
**K**	169.1	5.5	152.1	0.92	0.90
**L**	172.7	7.7	139.2	0.84	0.81
***Mean A-F, H-L (SD)***	*164.8 (10.7)*	*2.8 (6.7)*	*148.2 (5.3)*	*0.91 (0.03)*	*0.90 (0.05)*
**RECTUM**					
**Observer**	V_iPlan_ (cm^3^)	$\Delta V_{\mathrm{Obs}}^{\mathrm{iPan}}$ (%)	*V*_*Obs*∩*iPlan*_ (cm^3^)	*DSC*_*Obs*_^*iPlan*^	*I*_*Obs*_^*Obs*∩*iPlan*^
***iPlan***	*62.3*	*-*	*-*	*-*	*-*
**H**	49.6	−20.5	40.3	0.72	0.81
**A**	63.9	2.5	47.2	0.75	0.74
**I**	53.9	−13.5	45.0	0.77	0.84
**B**	60.4	−3.1	46.8	0.76	0.77
**C**	39.8	−36.2	31.1	0.61	0.78
**D**	58.0	−7.0	45.5	0.76	0.78
**E**	52.2	−16.3	46.1	0.80	0.88
**F**	51.2	−17.9	44.3	0.78	0.87
**J**	65.1	4.4	48.1	0.76	0.74
**K**	44.7	−28.3	35.1	0.66	0.79
**L**	67.6	8.4	52.2	0.80	0.77
***Mean A-F, H-L (SD)***	*55.1 (8.8)*	*−11.6 (14.1)*	*43.8 (6.1)*	*0.74 (0.06)*	*0.80 (0.05)*

^a^ Outline missing.

**Table 3:** Summary of quantitative measures for structures for the IR case benchmarking study patient

**CTV**					
**Observer**	V_iPlan_ (cm^3^)	$\Delta V_{\mathrm{Obs}}^{\mathrm{iPan}}$ (%)	*V*_*Obs*∩*iPlan*_ (cm^3^)	*DSC*_*Obs*_^*iPlan*^	*I*_*Obs*_^*Obs*∩*iPlan*^
***iPlan***	*28.5*	*-*	*-*	*-*	*-*
**A**	64.8	127.2	25.4	0.54	0.39
**B**	62.3	118.6	28.0	0.62	0.45
**C**	61.1	114.3	27.8	0.62	0.46
**D**	64.1	124.9	27.9	0.60	0.43
**E**	64.5	126.2	27.7	0.60	0.43
**F**	73.9	159.1	27.1	0.53	0.37
**G**	45.2	58.3	28.2	0.77	0.62
***Mean A-G (SD)***	*62.3 (8.6)*	*118.4 (30.2)*	*27.4 (1.0)*	*0.61 (0.08)*	*0.45 (0.08)*
**BLADDER**					
**Observer**	V_iPlan_ (cm^3^)	$\Delta V_{\mathrm{Obs}}^{\mathrm{iPan}}$ (%)	*V*_*Obs*∩*iPlan*_ (cm^3^)	*DSC*_*Obs*_^*iPlan*^	*I*_*Obs*_^*Obs*∩*iPlan*^
***iPlan***	*185.8*	*-*	*-*	*-*	*-*
**A**	186.2	0.2	168.1	0.90	0.90
**B**	181.7	−2.2	169.9	0.92	0.94
**C**	171.5	−7.7	160.9	0.90	0.94
**D**	-^a^	-^a^	-^a^	-^a^	-^a^
**E**	175.1	−5.8	162.5	0.90	0.93
**F**	-^b^	-^b^	-^b^	-^b^	-^b^
**G**	185.1	−0.4	172.1	0.93	0.93
***Mean A-G (SD)***	*179.9 (6.4)*	*−3.2 (3.4)*	*166.7 (4.8)*	*0.91 (0.01)*	*0.93 (0.01)*
**RECTUM**					
**Observer**	V_iPlan_ (cm^3^)	$\Delta V_{\mathrm{Obs}}^{\mathrm{iPan}}$ (%)	*V*_*Obs*∩*iPlan*_ (cm^3^)	*DSC*_*Obs*_^*iPlan*^	*I*_*Obs*_^*Obs*∩*iPlan*^
***iPlan***	*123.5*	*-*	*-*	*-*	*-*
**A**	152.6	23.5	106.3	0.77	0.70
**B**	112.1	−9.2	82.3	0.70	0.73
**C**	98.2	−20.5	86.9	0.78	0.88
**D**	100.6	−18.6	86.4	0.77	0.86
**E**	73.8	−40.3	64.3	0.65	0.87
**F**	118.7	−3.9	83.1	0.69	0.70
**G**	103.3	−16.4	84.7	0.75	0.82
***Mean A-G (SD)***	*108.5 (24.1)*	*−12.2 (19.4)*	*84.9 (12.2)*	*0.73 (0.05)*	*0.80 (0.08)*

^a^ Outline missing.

^b^ Structure not completely outlined.

**Figure 1:**
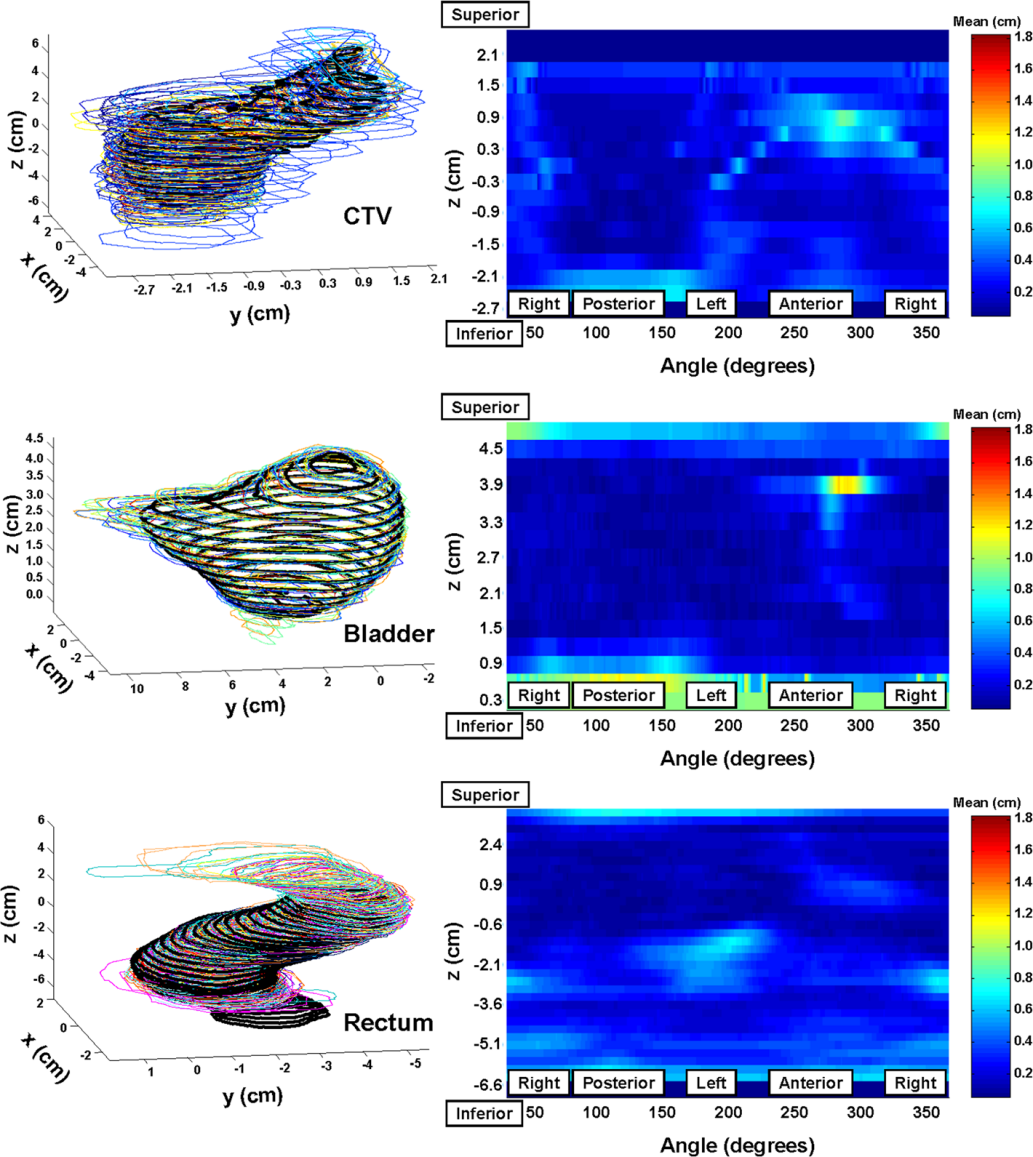
**Left – Multiple observer-defined structures for CTV, bladder and rectum for the HR patient, compared with the structures from iPlan (thick black lines).** Right - Surface maps of mean spatial differences between iPlan-segmented structures and observer-segmented structures.

**Figure 2:**
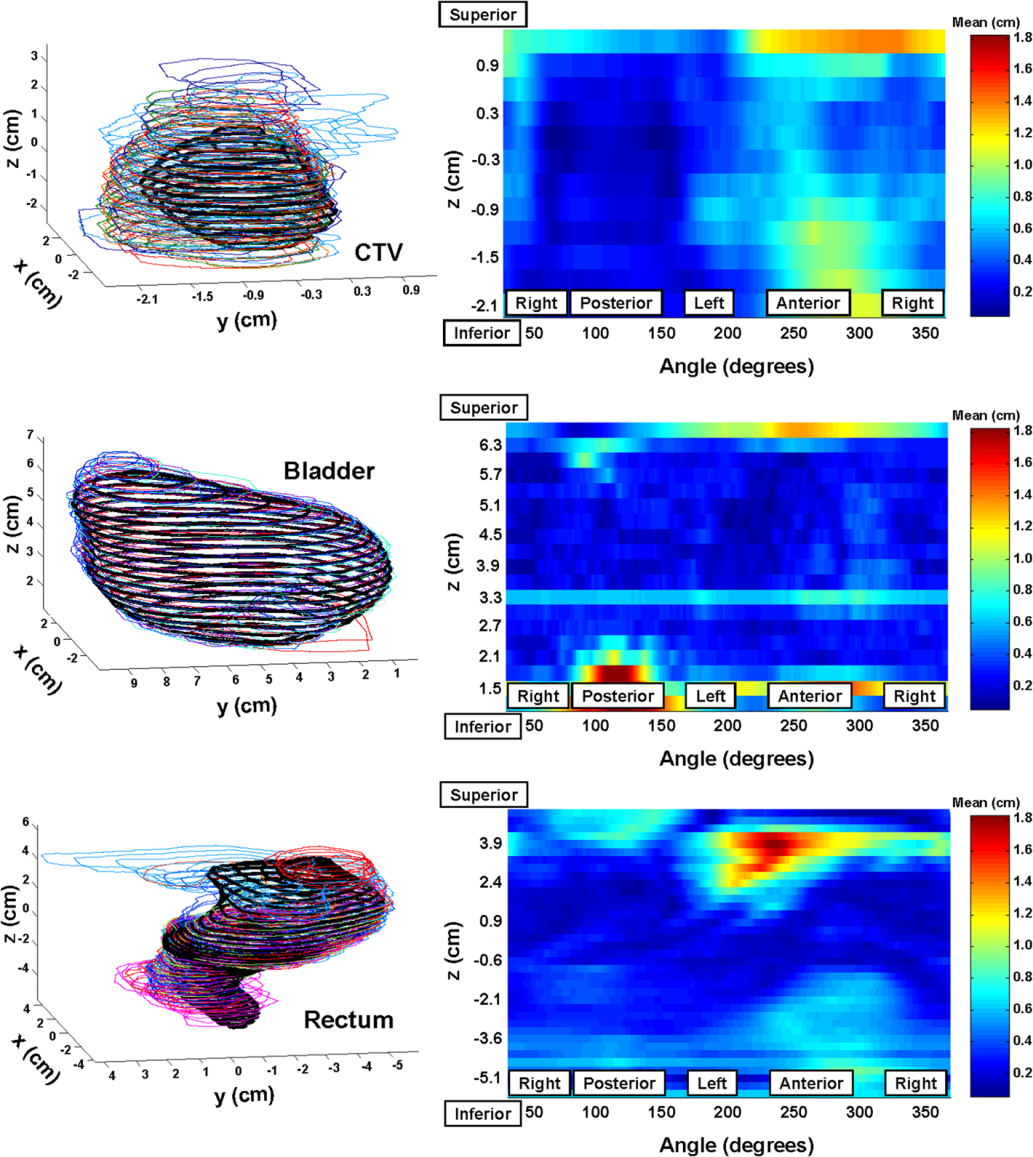
**Left – Multiple observer-defined structures for CTV, bladder and rectum for the IR patient, compared with the structures from iPlan (thick black lines).** Right - Surface maps of mean spatial differences between iPlan-segmented structures and observer-segmented structures.

In Figure 1 and 2, all observer contours for the three structures are shown overlayed in 3D views, providing some indication of the range of observer-defined structures relative to the iPlan-defined structures in each dimension. The quantitative spatial comparisons, referenced to the iPlan contours and projected onto the corresponding structure surface in each case, are provided on the right of Figures 1 and 2. For the HR case (Figure 1), one outlying CTV volume, due to observer J (see Table 2), was not included in the calculation of the represented statistic, being the mean distance between the iPlan-defined structure and each observer-defined structure. In these figures, regions with pixel colours higher up the ‘Mean’ scale indicate areas where observer-defined outlines depart more (spatially) relative to the iPlan-defined outlines. All scales have been adjusted to the maximum mean difference across the six colour-scaled figures. Note that the dark blue bands at the top and bottom of the map for the CTV, and bottom of the map for rectum in Figure 1 represent image slices where slices adjacent to an iPlan-defined contour did not have any observer-defined contours.

### Sample trial data

Table 4 provides quantitative comparison between the iPan-defined CTV, bladder and rectum for the 20 sample trial patients, and where available, the corresponding observer-defined structures. It should be remembered that in this case, these measures represent the mean and standard deviation of values derived for a maximum of 20 samples, each one involving a comparison of the iPlan-defined structure with that due to a single observer (being the clinician treating the corresponding patient). It is likely therefore that this data represents a comparison of the automatic segmentation routine against 20 independent observers, on different datasets.

**Table 4:** Summary of quantitative measures derived across the 20 sample trial patient datasets

	**CTV**	**BLADDER**	**RECTUM**
N	20	14	20
V_iPlan_ (cm^3^)	42.9 (14.0)	132.9 (79.7)	83.3 (24.7)
V_Obs_ (cm^3^)	77.6 (35.3)	121.2 (51.1)	120.1 (71.8)
$\Delta V_{\mathrm{iPlan}}^{\mathrm{Obs}}$ (%)^a^	91.4 (87.4)^b^	2.4 (26.5)	48.8 (86.3)^c^
*V*_*Obs*∩*iPlan*_ (cm^3^)	31.6 (12.52)	97.9 (48.9)	60.0 (19.6)
*DSC*_*Obs*_^*iPlan*^	0.53 (0.16)	0.77 (0.06)	0.62 (0.14)
*I*_*Obs*_^*Obs*∩*iPlan*^	0.46 (0.22)	0.77 (0.10)	0.74 (0.17)
*z*_*Sup*,*Obs*_ − *z*_*Sup*,*iPlan*_ (cm)	0.3 (1.2)	0.75 (0.89)	0.19 (1.87)
*z*_*Inf*,*Obs*_ − *z*_*Inf*,*iPlan*_ (cm)	−1.2 (1.0)	0.32 (1.13)	1.99 (1.02)

^a^ Relative to user-defined volume.

^b^ V_iplan_ and V_Obs_ values significantly different on a 2-tailed paired t-test at p=0.002.

^c^ V_iplan_ and V_Obs_ values significantly different on a 2-tailed paired t-test at p=0.03.

## Discussion

This study has provided evaluation of automatic segmentation, for one specific implementation, against expert observers for male pelvic anatomy in the context of a clinical trial. This has been for i) two patient datasets, each as assessed by multiple observers and the automatic segmentation routine, and ii) twenty datasets, each as assessed by a single observer and the automatic segmentation routine. The results from this study highlight:

The inherent variability in anatomical segmentation between experts.

Factors influencing the particular automatic segmentation technique investigated.

The limitations of the technique investigated for application in a clinical trial context.

They also lead to some recommendations regarding use of automatic segmentation in future multicentre trial QA and analysis.

Inter-observer inconsistencies in anatomical segmentation have multiple sources including the image contrast between the tissues of interest and the knowledge, experience and diligence of the observers. For both human observers and computational algorithms, it is apparent that agreement increases particularly as the contrast sensitivity of anatomical boundaries increase.

The automatic segmentation algorithm employed in the iPlan system does not rely purely on local image contrast features for automatically applying contours. Image deformation maps are first generated based on similar features between the whole patient and reference image sets. This allows inclusion of adjacent anatomical features in the consideration of deformation of an outline for any particular anatomy, in a similar way to that undertaken by a human observer.

The relatively distinct contrast at the bladder boundary is likely to lead to the consistency in bladder outlining seen for both the IR and HR cases, where DSC values are the highest of all structures for both the benchmarking cases and sample trial datasets. The bladder dominates image features in that region of the male pelvis which would result in local image registration that would substantially weight agreement of bladder structures and thus there is also consistency between iPlan-defined and the observer-defined structures, and the corresponding total bladder volumes in Table 3. The principal region of disagreement is at the inferior border of the bladder where interpretation of the bladder/prostate interface could be contentious. The mean positive values of *z*_*Sup*,*Obs*_ − *z*_*Sup*,*iPlan*_ and *z*_*Inf*,*Obs*_ − *z*_*Inf*,*iPlan*_ from Table 4 indicate the tendency of observers to place outlines on more superior slices to iPlan.

For rectum, reasonable agreement is seen for with the benchmarking cases for observers and iPlan over the range of slices covered by the iPlan-defined structure, except for a small region for the HR patient (see Figure 2) where the iPlan contours extended to the left/anterior of the rectum identified by observers. There appears to be no distinct feature of the HR-patient CT set to cause this. Without access to the reference images used in the iPlan atlas we can only speculate on anatomical differences between the reference images and HR-patient images that might have caused this. Otherwise, there are substantial differences in rectal outlines at the superior/inferior ends due to differences in interpretation of extent of the rectum, particularly towards the anal canal (revealed by the large positive mean *z*_*Inf*,*Obs*_ − *z*_*Inf*,*iPlan*_ shown for rectum in Table 4). The definition of the inferior extent of rectum for the RADAR trial was tied to the location of the prostatic apex (see Table 1). Observers submitting RADAR trial data included considerably more of the anterior region extending into bowel which has skewed results for the rectal volumes considerably – this difference is the principal reason for volume discrepancies seen in Tables 2, 3, 4. Otherwise, the relatively high values of *I*_*Obs*_^*Obs*∩*iPlan*^ for rectum across Tables 2, 3, 4 indicate significant inclusion of the iPlan-defined rectal volume by observers. It should be noted that QA during the RADAR trial highlighted the variable interpretation of superior extent of the rectum between submitting clinicians [13].

In terms of prostate volume agreement, there is some contrast in the agreement achieved with the IR case (Figure 1) and the HR case (Figure 2). The relatively low values of *DSC*_*Obs*_^*iPlan*^ and *I*_*Obs*_^*Obs*∩*iPlan*^ (across all of Tables 2 to 4) indicate that not only were the iPlan derived CTVs significantly smaller than those of the observers’, but that there were frequently regions of disagreement, particularly for the IR case. For the HR case, whilst most observers segmented a structure larger than that automatically segmented, one particularly generous observer’s segmented structure (observer J in Table 2) eclipses the others substantially, dominating results. The principal region for disagreement for CTV was at the prostatic apex, a well-recognised location for observer-disagreement when using CT imaging [7,9,10], where most observers included more inferior slices to that from the automatic segmentation. During the RADAR trial, an ‘audit’ of CTV outlining was undertaken where definition of the prostatic apex proved highly variable, with many contributing clinicians extending prostate definition far more inferiorly than considered acceptable by RADAR investigators [13]. This is consistent with the results presented here, should the iPlan-defined CTV be considered representative of expert opinion, where (as shown in Table 4) the observer derived CTVs extended on average 1.2 cm more inferiorly. For the HR case there was also some disagreement at the sharp gradient where the prostatic base joins the seminal vesicles.

For the IR benchmarking case, automatic segmentation generated a prostate volume that was much smaller than that from the observers, in all directions except posteriorly at the rectal border. However, there was quite good agreement amongst the observers themselves. Given the better agreement between observers and automatic segmentation for the HR case, it is hypothesised that differences in local anatomy (relative to the iPlan reference images) as identified in the IR-case images led to the generation of a considerably smaller prostate volume. The IR-case patient was dimensionally smaller (left-right and anterior-posterior separation at level of prostate centre 35.9 cm and 19.6 cm respectively) than the HR-case patient (40.6 cm and 25.1 cm respectively). There is some influence of image quality on the registration obtained in iPlan [16], though both sample patients were imaged on the same CT scanner with the same X-ray tube settings and at the same resolution.

The automatic segmentation technique investigated here, whereby a reference image and volume set is adapted to a new dataset, relies heavily on the anatomical definitions used in creating the reference structures. Translating those volumes to multiple datasets collected during a trial will systematically transfer that individual definition. It is hypothesized that a technique that utilizes a training set of patient data, with volumes defined via several investigators, and whereby volumes are defined by mapping contours onto patient-specific features of image data [21-23], could alleviate the resulting systematic bias.

## Conclusions

This study has demonstrated the utility of quantification of segmented volumes for evaluating and comparing observer and automatically-defined anatomical volumes in the context of a clinical trial. The variability seen stems from a combination of patient-specific imaging features, variable contrast between particular organs and surrounding anatomy, the nature of the automatic segmentation technique investigated and the variability between experts.

When implementing automatic segmentation software it is important to acknowledge that the resulting structures will inherently depend on the limited data used to define reference anatomy (ie., limited in patient numbers and limited in observers) and the potential discrepancy with the opinion and/or experience of the user. Where the algorithm used for mapping contours depends on a corresponding image registration algorithm, that discrepancy will also likely have inter-patient variability as observed here for prostate definition. This variability is likely to increase even further if poorer quality images are being used, such as those derived using cone-beam CT methods.

### Endnotes

^a^ Note that the terms ‘contour’, ‘outline’, and ‘volume’ are frequently used interchangeably to describe regions of interest on radiographic images obtained by the method of ‘segmentation’, ‘contouring’, ‘outlining’, ‘voluming’ or ‘delineation’, with the resulting regions of interest interchangeably called ‘contours’, ‘volumes’ and ‘structures’. Here we refer to a ‘structure’ as the 3D object constituted by a series of individual 2D ‘contours’ obtained by ‘segmentation’ of regions of interest on an individual patient’s radiographic images.

^b^ Note the convention used here is X axis is left-right, Y axis is anterior-posterior, and Z axis is superior-inferior.

## Competing interests

The authors declare that they have no competing interests.

## Authors’ contributions

JPG collated treatment plan data, imported plans into iPlan for automated segmentation, processed treatment plans to extract structures, and collated structure volume data. GG assisted with import of plans into iPlan and subsequent re-export and interpretation of data. MAE was involved in design and implementation of the technical quality assurance of the RADAR trial, the design, development and use of the SWAN and VAST software systems, the derivation of spatial statistics and data interpretation. All authors were involved in the preparation and review of the manuscript. All authors read and approved the final manuscript.
